# Advancing Rice Grain Impurity Segmentation with an Enhanced SegFormer and Multi-Scale Feature Integration

**DOI:** 10.3390/e27010070

**Published:** 2025-01-15

**Authors:** Xiulin Qiu, Hongzhi Yao, Qinghua Liu, Hongrui Liu, Haozhi Zhang, Mengdi Zhao

**Affiliations:** 1 School of Automation, Jiangsu University of Science and Technology, Zhenjiang 212100, China; qiuxiulin@njust.edu.cn; 2 School of Computer, Jiangsu University of Science and Technology, Zhenjiang 212100, China; 221210701227@stu.just.edu.cn (H.Y.); 231210703115@stu.just.edu.cn (H.L.); 221210701230@stu.just.edu.cn (H.Z.); 3 College of Materials Science and Engineering, Suzhou University of Science and Technology, Suzhou 215011, China

**Keywords:** rice, impurities, semantic segmentation, SegFormer

## Abstract

During the rice harvesting process, severe occlusion and adhesion exist among multiple targets, such as rice, straw, and leaves, making it difficult to accurately distinguish between rice grains and impurities. To address the current challenges, a lightweight semantic segmentation algorithm for impurities based on an improved SegFormer network is proposed. To make full use of the extracted features, the decoder was redesigned. First, the Feature Pyramid Network (FPN) was introduced to optimize the structure, selectively fusing the high-level semantic features and low-level texture features generated by the encoder. Secondly, a Part Large Kernel Attention (Part-LKA) module was designed and introduced after feature fusion to help the model focus on key regions, simplifying the model and accelerating computation. Finally, to compensate for the lack of spatial interaction capabilities, Bottleneck Recursive Gated Convolution (B-$g^{n}$Conv) was introduced to achieve effective segmentation of rice grains and impurities. Compared with the original model, the improved model’s pixel accuracy (PA) and F1 score increased by 1.6% and 3.1%, respectively. This provides a valuable algorithmic reference for designing a real-time impurity rate monitoring system for rice combine harvesters.

## 1. Introduction

Rice is one of the world’s major staple crops, accounting for 12% of global arable land and providing food for over 50% of the population [1,2,3]. The impurity rate in paddies is an important indicator of rice quality [4]. During the harvesting and processing of rice, various impurities are often mixed in. A high impurity rate can reduce the quality of processed products, affecting the taste and appearance of the food. In international trade, the impurity rate of paddies is also an important trade standard. Most countries have specific restrictions and regulations regarding the impurity rate of imported rice. Understanding and controlling the impurity rate helps agricultural exporters and government agencies comply with international trade standards, ensuring product quality and compliance.

Rice impurities significantly affect the quality of rice grains and impede the efficiency of processing systems. Improving the segmentation accuracy of rice grains and impurities is, therefore, an important task in rice sorting and quality control. Currently, modern rice-sorting systems, such as optical sorters and pneumatic separators [5,6], are widely adopted in industrial rice processing. Optical sorters utilize high-resolution cameras to classify rice grains based on color, size, and shape, offering high sorting speed and accuracy. However, they often face challenges such as poor lighting, dust interference, or occlusion of impurities, which significantly affect their performance. Pneumatic separators, on the other hand, separate impurities based on density differences. Although effective in some scenarios, they struggle to distinguish impurities with similar densities to rice grains. These shortcomings highlight the need for advanced segmentation methods to address challenges, such as occlusion, adhesion, and detecting small impurities.

Moreover, advances in 3D vision systems offer greater potential for precise and fast detection of target regions. For example, Sergiyenko and Tyrsa [7] developed a 3D optical machine vision sensor with intelligent data management, which significantly improved robotic swarm navigation performance and enhanced 3D mapping capabilities. Similarly, Ivanov et al. [8] demonstrated the effectiveness of 3D data cloud fusion technology in improving the autonomous navigation accuracy of robotic groups in unknown terrains. Additionally, Sergiyenko et al. [9] proposed a synchronized data transfer model that effectively optimized obstacle detection and navigation. While these technologies have shown remarkable results in robotics, their application to agricultural tasks, such as rice impurity segmentation, remains largely unexplored. The advancements in 3D vision systems provide potential directions for achieving more precise segmentation and positioning in complex agricultural environments through the integration of multi-modal information.

This study focuses on improving the segmentation accuracy of rice grains and impurities using a 2D image-based approach. In recent years, advances in computer vision and deep learning technologies have provided new solutions for automatically detecting and classifying crop impurities, significantly improving efficiency and accuracy. Deep learning algorithms can learn complex feature patterns from a large amount of image data, thereby accurately classifying different types of impurities. This automation not only improves work efficiency but also reduces the need for manual intervention [10].

Based on deep learning, semantic segmentation algorithms can be divided into two main categories: methods based on convolutional neural networks (CNNs) and methods based on Transformers. The Fully Convolutional Network (FCN) [11] is one of the earliest proposed semantic segmentation algorithms based on CNNs. The FCN replaces traditional fully connected layers with fully convolutional layers, achieving end-to-end pixel-level classification.

Convolutional neural networks (CNNs) have dominated semantic segmentation in agriculture. Representative research includes the following: Jin et al. [12] proposed an intelligent detection method for mechanized soybean harvesting quality based on an improved U-Net algorithm, using the VGG16 network as the feature extraction module. The system’s evaluation indices for recognizing intact soybeans, broken soybeans, and impurities were 93.04%, 89.40%, and 96.49%, respectively. Compared with manual detection, the maximum absolute error for detecting soybean breakage rate was 0.57%, and for impurity rate, it was 0.69%.

Liu et al. [13] proposed a lightweight fully convolutional rice impurity segmentation algorithm based on deep learning, using an improved EfficientNetV2 network model and introducing a Normalized Attention Mechanism (NAM) to enhance feature extraction performance. The average detection time for a single image was 0.103 s on GPU devices and 0.301 s on CPU devices, demonstrating the lightweight nature of the algorithm.

However, CNNs have several drawbacks, such as limited receptive fields and the inability to capture global information, which significantly reduces their segmentation accuracy in impurity detection [14,15]. The core self-attention mechanism of Transformers can capture long-range information and dynamically adjust the receptive field according to the image content [16]. Therefore, Transformers exhibit stronger performance and flexibility compared to CNNs. Vision networks based on Transformers have already been researched in agriculture. For example, Yang et al. [17] proposed a new model called ECA-SegFormer, which enhances feature representation robustness by introducing the Efficient Channel Attention (ECA) module and Feature Pyramid Network (FPN) into the SegFormer decoder. ECA-SegFormer achieved an average pixel accuracy of 38.03% and an average intersection over union (IoU) of 60.86% on the dataset.

SegFormer [18], as a Transformer-based visual recognition network, offers better computational efficiency and feature extraction capabilities. However, it has issues such as insufficient feature utilization and an overly simplistic decoder. In this paper, we take the SegFormer backbone as the feature extractor and redesign the decoder.

The main contributions of this paper are as follows:

1. To address the problem of insufficient feature utilization, we use an optimized Feature Pyramid Network (FPN) to replace the original MLP layer, enhancing the semantic information of features;
2. A novel attention module (Part-LKA) was designed, which can independently adjust attention for different parts, enhancing the model’s focus on important features;
3. Bottleneck Recursive Gated Convolution (B-$g^{n}$Conv) was designed based on Recursive Gated Convolution ($g^{n}$Conv) [19] to reduce training costs and improve the network’s spatial interaction capabilities;
4. Different models were trained on a self-built dataset, and their performance was verified using a test set. The results show that the proposed method achieved higher accuracy in rice impurity segmentation, demonstrating its effectiveness.

## 2. Improved SegFormer Network Architecture

**Figure 1:**
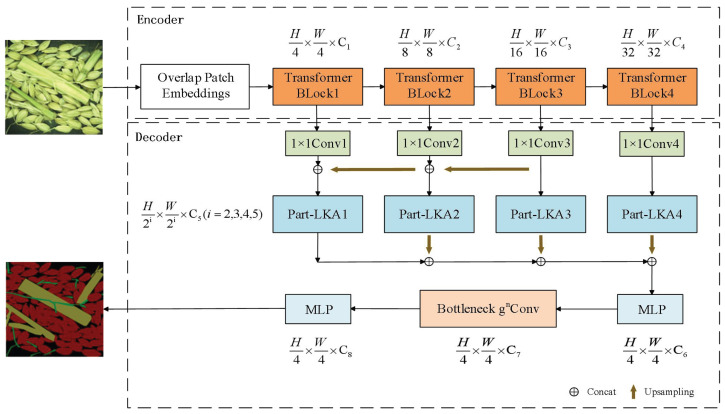
Improved model network architecture. Note: H represents the height (number of rows of pixels), W represents the width (number of columns of pixels), C represents the number of channels, MLP stands for Multi-Layer Perceptron, Part-LKA represents the Part Large Kernel Attention module, and B-$g^{n}$Conv represents the Bottleneck Recursive Gated Convolution.

The improved SegFormer network model mainly consists of two parts: an encoder and a decoder, with the overall structure illustrated in Figure 1. The encoder is responsible for extracting multi-scale features, while the decoder aggregates and processes these multi-scale features to generate the final image output.

The encoder’s input module resizes the input image to a uniform pixel size, and the Transformer Block processes it to produce feature maps at different resolutions. First, an optimized Feature Pyramid Network (FPN) is utilized to combine high-level semantic features with low-level features, generating richer feature maps. Subsequently, the Part-Large Kernel Attention (Part-LKA) operation is applied to the fused features, allowing the network to focus on specific dimensions, thus enhancing feature relevance and better handling of local contexts. The attention-adjusted features are then upsampled using bilinear interpolation and resized to 1/4 of the input image size for dimensional concatenation. Next, two 1 × 1 convolutions are used to adjust the channel count to effectively fuse features from different scales. Additionally, Recursive Gated Convolution ($g^{n}$Conv) is integrated into the 1 × 1 convolutions to enhance spatial interaction within the network and capture hierarchical features. The final output is the decoded semantic segmentation map.

### 2.1. Encoder

**Figure 2:**
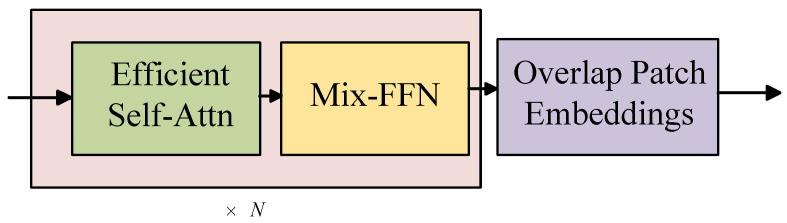
Encoder network architecture.

The encoder adopts the MiT-B0 structure from the SegFormer model, which is composed of four Transformer Blocks, as shown in Figure 2. Overlap Patch Embeddings (OPE) are used for feature extraction and downsampling of the image. The standard convolution layer is used to scale the feature map by modifying the patch size and stride, ensuring that patches overlap, thereby establishing connections and converting two-dimensional features into one-dimensional features. Next, Efficient Self-Attention (ESA) and a Mix Feed-Forward Network (Mix-FFN) are employed for self-attention computation and feature enhancement. Additionally, to extract richer details and semantic features, the Transformer Block uses multiple stacked ESAs and Mix-FFNs to increase the network depth.

ESA (Efficient Self-Attention) is similar to the traditional self-attention mechanism in structure, but it employs a sequence reduction operation to reduce computational complexity. The principle of the traditional self-attention mechanism is shown by Equation (1) as follows:

(1) $$\mathrm{Attention}\left(Q,K,V\right)=\mathrm{Softmax}\left(\frac{QK^{⊤}}{\sqrt{d_{\mathrm{head}}}}\right)V$$

where *Q*, *K*, and *V* are all N×C matrices, and *N* represents the sequence length H×W. By performing a dot product between *Q* and *K*, the similarity between feature maps is calculated to obtain the attention scores. These scores are then multiplied with the original feature map *V* to extract data. At this point, the computational complexity of the self-attention mechanism is $O(N^{2})$, which is not favorable for large images. Therefore, a sequence reduction factor *R* is used to shorten the sequence, with the specific operation as follows:

(2) $$\hat{K}=Reshape\left(\frac{N}{R},C,R\right)\left(K\right)$$

(3) $$K=Linear\left(C\cdot R,C\right)\left(\hat{K}\right)$$

where *N* represents the number of heads in the self-attention mechanism, and *R* represents the scaling factor for each self-attention mechanism. After processing, the resulting matrix size is $\frac{R}{N}\times C$.

Mix-FFN uses a 3×3 convolution to dynamically express the inter-patch relationships, thereby replacing the fixed positional encoding used in ViT [20]. By placing convolution within the FFN, the impact of zero-padding on positional encoding is reduced. The specific operation is as follows:

(4) $$X_{\mathrm{out}}=MLP\left(\mathrm{GELU}\left({\mathrm{Conv}}_{3\times 3}\left(MLP\left(X_{\mathrm{in}}\right)\right)\right)\right)+X_{\mathrm{in}}$$

where $X_{\mathrm{in}}$ represents the features derived from the attention mechanism.

### 2.2. Optimization of FPN Structure

**Figure 3:**
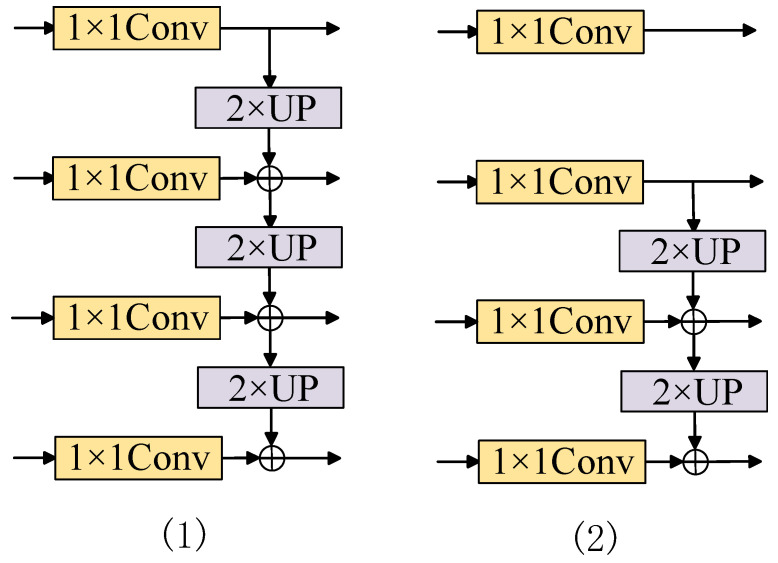
FPN structure before and after improvement.

The FPN structure used in the decoder was proposed by Lin et al. [21], and it effectively detects objects of different sizes, improving detection accuracy and robustness. In the field of semantic segmentation, FPN provides rich contextual information, which is crucial for accurately segmenting small objects or complex details in images, as well as for improving object boundary handling, as shown in Figure 3(1).

For smaller targets, shallower features are more beneficial for segmenting fine details, while incorporating only a part of the high-level features into the lower-level features is favorable for the upsampling process to restore image resolution. Therefore, the FPN structure was optimized. Experimental comparisons show that directly outputting the highest-level features while fusing other features using FPN maximizes the retention of detailed information. This improvement enhances the model’s accuracy while maintaining its original advantages. The structure is illustrated in Figure 3(2).

### 2.3. Part Large Kernel Attention Module

**Figure 4:**
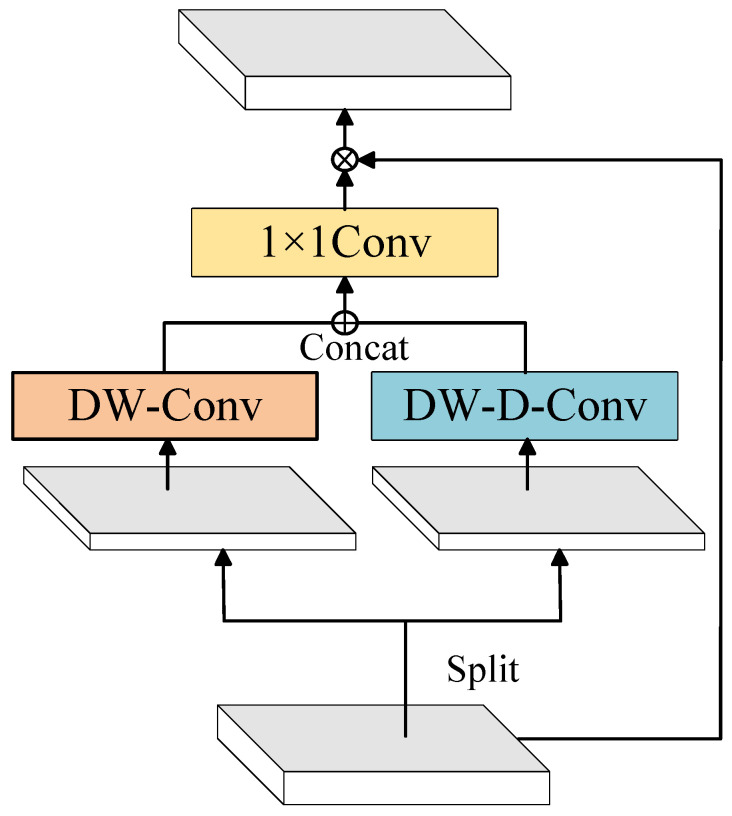
Part-LKA network structure.

The original SegFormer uses Multi-Layer Perceptrons (MLPs) for simple feature transformation. Although this approach reduces the computational complexity of the model, it is unable to effectively filter important feature information during the transformation process. To extract significant feature information, this paper proposes the Part Large Kernel Attention (Part-LKA) module, with the network structure illustrated in Figure 4. The proposed Part-LKA considers both channel and spatial dimensions simultaneously.

The input feature map is divided into two parts along the channel dimension, and depthwise convolution and dilated depthwise convolution are performed separately. Depthwise convolution is applied independently on each channel to capture spatial features within each channel, without mixing information between different channels. The dilated depthwise convolution, with a dilation rate of 3, further expands the receptive field to capture more extensive spatial features. The feature maps obtained by depthwise convolution and dilated depthwise convolution are concatenated along the channel dimension, thus integrating spatial information at different scales. The 1 × 1 convolution used in the model not only fuses channel information but also captures the relationships between different channels. The generated attention map is then multiplied elementwise with the original input feature map to dynamically adjust the feature intensity at each spatial position and across channels, thereby emphasizing important spatial locations and channel features. The specific operations are as follows.

(5) $$F=F_{1}⊕F_{2}$$

(6) $$\mathrm{Attention}={\mathrm{Conv}}_{1\times 1}\left(DW\cdot D\cdot \mathrm{Conv}\left(F_{1}\right)⊕DW\cdot \mathrm{Conv}\left(F_{2}\right)\right)$$

(7) Output=Attention⊗F

where ⊕ represents concatenation along the feature dimension, $F_{1}$ and $F_{2}$ are matrices of size H×W×C/2, and ⊗ denotes elementwise multiplication.

### 2.4. Bottleneck Recursive Gated Convolution

Recursive Gated Convolution ($g^{n}$Conv) enhances the representation capability of convolutional neural networks by recursively applying convolution operations while controlling the flow of information. The growth environment of rice is complex, involving impurities of various types and shapes, which poses a challenge for accurate segmentation. Traditional convolution methods usually apply fixed weights to all input positions, ignoring the uniqueness of local image regions, resulting in the inability to accurately identify impurity features in complex scenarios. Therefore, a single convolution operation is very limited in handling such fine-grained visual tasks.

To address these issues, this study introduces the $g^{n}$Conv module. The goal of the $g^{n}$Conv module is to achieve long-range modeling and high-order spatial interaction. It is constructed using standard convolutions, linear projections, and elementwise multiplication, but has input-adaptive spatial mixing functionality similar to self-attention. In CNNs, networks mainly use static convolution kernels to aggregate neighboring features, whereas Vision Transformers use multi-head self-attention (MSA) to dynamically aggregate spatial token weights. However, the quadratic complexity and large input size of self-attention significantly limit the application of Vision Transformers. In contrast, $g^{n}$Conv achieves equivalent spatial interaction using simple operations such as fully connected layers of convolutional kernels. The basic module of this method is gated convolution. Let $x\in R^{HW\times C}$ be the input feature of the gated convolution, then the output *y* can be represented as:

(8) $$\left[\begin{array}{c} p_{0}^{HW\times C},q_{0}^{HW\times C} \end{array}\right]=\phi \left(x\right)\in R^{HW\times 2C}$$

(9) $$p_{1}=f\left(q_{0}\right)⊙p_{0}\in R^{HW\times C}$$

(10) $$y=\phi \left(p_{1}\right)\in R^{HW\times C}$$

where the input *x* is linearly projected and then split into channels to obtain $p_{0}$ and $q_{0}$; the function f() represents the computation through depthwise convolution, and ϕ denotes the linear projection.

Through multiple recursive convolution processes, in each recursion, the input features are convolved with depthwise convolution kernels, and the resulting output is combined elementwise with the output of pointwise convolution. This gating mechanism enables the model to selectively retain important information or discard irrelevant data based on specific context, thereby managing the flow of information more effectively and capturing hierarchical features within paddy images.

**Figure 5:**
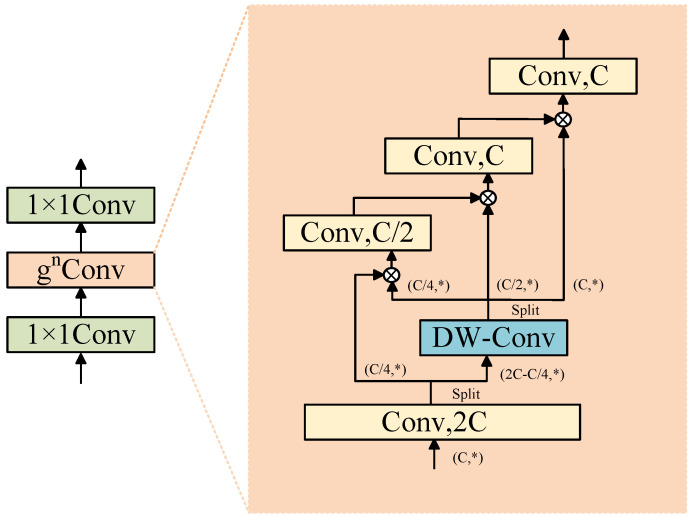
Bottleneck-$g^{n}$Conv network structure.

To achieve a balance between computational cost and representational power, this module is combined with two 1 × 1 convolutions to reduce the model’s parameter count and computational complexity, forming what is termed the Bottleneck Recursive Gated Convolution (B-$g^{n}$Conv) module, as shown in Figure 5. In the initial stage, feature compression reduces the computational burden, followed by recursive gated convolution to capture complex spatial hierarchies, and the extracted features are then remapped to a higher-dimensional space for further processing. This process not only enhances the model’s ability to learn details but also mitigates accuracy loss caused by input feature compression.

## 3. Experiments

### 3.1. Dataset

**Figure 6:**
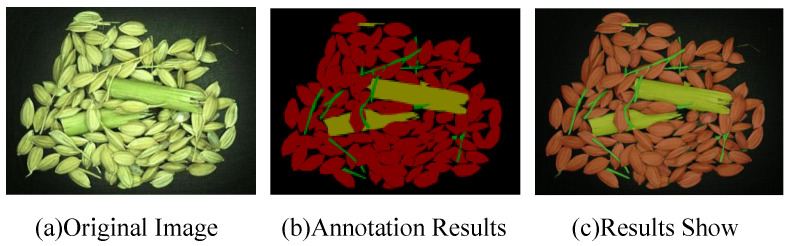
Image annotation results.

The rice impurity sample images were captured using a Huashi LRCP10230 industrial camera with a lens focal length of 12 mm. Under an LED light source (model: YSC-R9060_W, manufactured by YVSION in Shenzhen, China), the light source was positioned at a 45-degree angle to minimize shadows and ensure uniform illumination. The industrial camera was used to sample the rice impurity samples in the sampling box of the harvester. A total of 4288 images with a resolution of 800 × 600 were taken and saved in JPG format. The LabelImg tool was used to annotate the rice grains and impurities, with the annotations categorized into four classes: rice grains, stems, branches, and background. Each of the four categories, including the background, was marked with specific RGB values: rice grains [128, 128, 128], stems [0, 128, 0], branches [128, 0, 0], and background [0, 0, 0]. The image annotation process is shown in Figure 6.

Before training the network, the 4288 labeled rice impurity images were divided into training, validation, and test sets in a ratio of 8:1:1. Additionally, to prevent model overfitting and improve robustness, data augmentation techniques, such as random flipping, contrast enhancement, Gaussian blur, grayscale processing, and brightness enhancement, were applied during the training process.

### 3.2. The Model Training Environment

The improved algorithm was trained and tested using the deep learning framework PyTorch on a desktop computer, with the hardware and software parameters shown in Table 1. For the hardware setup, the computer used an AMD Ryzen 5 5600G processor (CPU) (Advanced Micro Devices, Inc., Santa Clara, CA, USA) and an NVIDIA GeForce GTX 1660s graphics card (GPU) (NVIDIA Corporate, Santa Clara, CA, USA) with 6 GB of video memory. For the software environment, the operating system was Windows 10, Python version 3.8, PyTorch framework version 1.12, and CUDA 11.6 was utilized for acceleration.

The input size of the model was set to 512 × 512, with a batch size of 8. The optimizer selected was Adam, with an initial learning rate of $1\times 10^{-4}$, using a cosine decay schedule, and the minimum learning rate was set to 0.01 times the initial learning rate. The weight decay parameter was set to 0.01, and the momentum factors Beta1 and Beta2 were set to 0.9 and 0.999, respectively, for first-order and second-order moment estimation. The dropout ratio was set to 0.1 to prevent overfitting, and the convolutional kernel size was set to 3. The loss function was a combination of cross-entropy loss and Dice loss for gradient calculation. The number of training epochs was set to 800 to ensure sufficient convergence of the model.

### 3.3. Experimental Evaluation Metrics

To properly evaluate the proposed method, model parameter count and computational complexity were taken as key metrics, combined with pixel accuracy (PA), class pixel accuracy (CPA), mean intersection over union (MIoU), and comprehensive evaluation (F1) to assess model performance. The mathematical expressions for calculating PA, CPA, MIoU, and F1 are as follows:

(11) $$\mathrm{PA}=\frac{\mathrm{TP}+\mathrm{TN}}{\mathrm{TP}+\mathrm{TN}+\mathrm{FP}+\mathrm{FN}}$$

(12) $$\mathrm{CPA}=\frac{\mathrm{TP}}{\mathrm{TP}+\mathrm{FP}}$$

(13) $$\mathrm{mIoU}=\frac{\mathrm{TP}}{\mathrm{TP}+\mathrm{FN}+\mathrm{FP}}$$

(14) $$F_{1}=\frac{{2\mathrm{TP}}^{2}}{2\mathrm{TP}+\mathrm{FN}+\mathrm{FP}}$$

where TP represents the pixel correctly classified as belonging to the target class, TN represents the pixel correctly classified as not belonging to the target class, FP represents the pixel incorrectly classified as belonging to the target class when it does not, and FN represents the pixel incorrectly classified as not belonging to the target class when it does.

### 3.4. Performance Comparison of Various Semantic Segmentation Models

To objectively evaluate the performance of the improved model in segmenting rice impurities, the proposed model was compared with the original model and several mainstream models under the same configuration and initial training parameters. The results are shown in Table 2.

As shown in Table 2, compared to the original model, the improved model achieved increases of 1.6%, 5.06%, and 3.1% in PA, MIoU, and F1, respectively. Compared to other mainstream models, the improved network achieved the best accuracy for all metrics, significantly outperforming other networks. In terms of model lightweighting, the improved model’s parameter count is only 4.07 M, which is 16.53 M, 42.64 M, 20.82 M, 1.72 M, and 5.57 M fewer than NAM-EfficientNetv2, PSPNet [22], U-Net [23], DeepLabV3+ [24], and HRNet [25], respectively. Additionally, the computational complexity of the improved model is 4.66 G, which is 18.05 G, 26.24 G, 108.42 G, 8.56 G, and 4.71 G lower than those of the aforementioned networks, respectively.

### 3.5. Performance Comparison of Different Attention Modules

To evaluate the effectiveness of the Part-LKA module designed in this study for improving accuracy, we conducted a series of experiments. The Part-LKA module was replaced in the same position within the model with five major modules: the Efficient Multi-Scale Attention Module (EMA) [26], Coordinate Attention (CoordAtt) [27], Squeeze-and-Excitation Network (SE) [28], Efficient Channel Attention (ECA) [29], and Convolutional Block Attention Module (CBAM) [30]. The comparison results are shown in Table 3.

As shown in Table 3, it is evident that, after replacing the SE and ECA modules, the model’s parameter count and computational complexity decreased, but this was accompanied by a corresponding decline in accuracy. Compared to SE and ECA, Part-LKA improved PA by 1.43% and 1%, respectively, increased MIoU by 4.61% and 3.34%, respectively, and enhanced F1 by 2.82% and 2.03%, respectively. Moreover, compared with EMA, CoordAtt, and CBAM, the Part-LKA module achieved increases in PA of 0.68%, 1.46%, and 1.27%, respectively, and improvements in F1 of 1.37%, 2.79%, and 2.43%, respectively, while having a lower parameter count and computational complexity. Therefore, Part-LKA demonstrates superior feature selection ability for identifying impurity features, surpassing the compared attention modules, effectively improving the model’s performance in rice grain and impurity segmentation.

### 3.6. Performance Comparison of Different Feature Fusion Modules

To verify the issue of insufficient feature utilization, we designed a comparative experiment to evaluate the impact of different feature fusion modules on the performance of the SegFormer network. Table 4 shows the performance comparison between the original model (NONE) and three feature fusion modules (FPN, U-Net, SFM [31]), where GPA represents the pixel accuracy of rice grains, SPA represents the pixel accuracy of impurity stems, and BPA represents the pixel accuracy of impurity branches. The original model only performs simple dimensional adjustments and upsampling of features through the MLP layer. The results show that its PA is 96.17%, F1 is 88.47%, and it performs poorly in SPA and BPA, which are 79.8% and 83.6%, respectively. This indicates that the original model has significant shortcomings in multi-scale feature fusion and fails to fully extract fine-grained semantic information.

In contrast, after using the FPN module, although the PA increased slightly to 96.18%, the precision of the SPA and BPA improved to 79.74% and 83.78%, respectively, and the F1 score increased to 88.49%. This validates that the top-down feature fusion mechanism of the FPN can effectively integrate multi-scale information, thereby enhancing the model’s ability to express target features. On the other hand, U-Net and SFM resulted in performance degradation due to their less suitable feature fusion methods, especially with SFM, where SPA and BPA dropped to 64.87% and 78.15%, respectively. These results further highlight the impact of insufficient feature utilization. Through this experiment, we confirmed the issue of insufficient feature utilization in the original model and demonstrated the advantages of the FPN module in improving network performance.

### 3.7. Visualization Analysis

**Figure 7:**
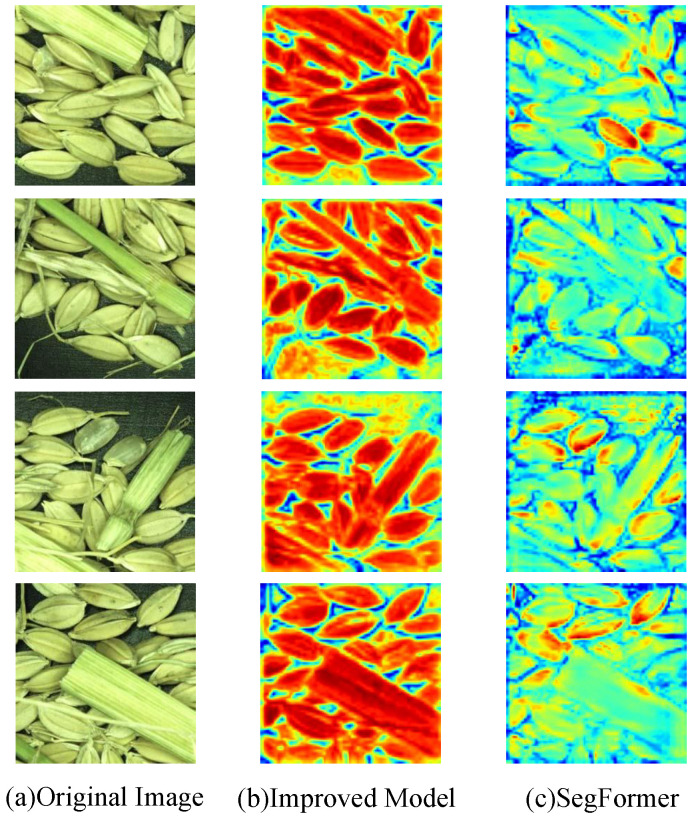
Class Activation Maps of sample images from the original and improved models.

To better perform a qualitative analysis of the model, complex rice grain images containing impurities were selected from the test set as samples. By calculating weights using the global average of the gradients, these weights can be used to weight the feature maps, generating a Class Activation Map (CAM) to observe the importance of each pixel for the classification results. SegFormer and the improved model generated CAMs in the last layer, as shown in Figure 7. Subfigure (a) shows some sample images from the test set, Subfigure (b) shows the CAMs generated by the improved model, and Subfigure (c) shows the CAMs generated by the original model.

As seen in Figure 7, the CAMs generated by SegFormer show limited attention to the grains and impurities, with blurred boundaries between different targets. In contrast, the CAMs generated by the improved model focus more on the rice grains and impurities, and compared to SegFormer, the contours between different targets are more distinct, providing clearer segmentation.

**Figure 8:**
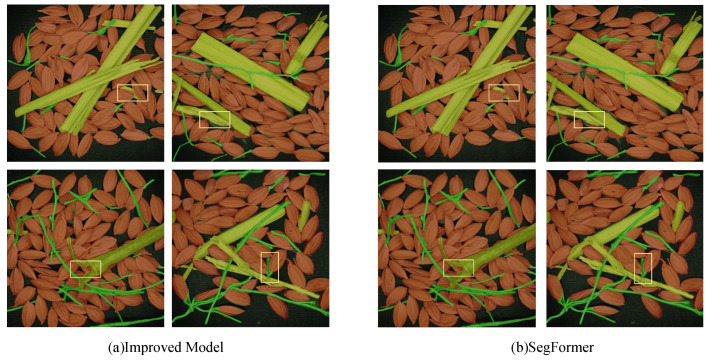
Visual comparison of segmentation results between the original and improved models.

Finally, the segmentation effect of the model on the original images was visualized with post-processing, and the original images were also visualized using SegFormer. Partial results of the original image processing are shown in Figure 8. It can be seen that, compared to the rough and uneven boundaries of the original model, the improved model predicts boundaries more clearly and smoothly, and for impurities at the same locations, the improved model has higher accuracy in segmentation. This indicates that the improved model has good potential for practical applications.

### 3.8. Performance Comparison of Ablation Experiments

To understand the contribution of each module in the improved decoder to the overall model performance, corresponding ablation experiments were designed. The results of the ablation experiments are shown in Table 5.

As shown in Table 5, four different ablation comparison experiments were designed. The first group used SegFormer for testing, while the second group replaced the MLP layer of the original model with an improved FPN structure in the decoder. The last two groups progressively added the Part-LKA and B-$g^{n}$Conv modules. By comparing the performance metrics of the models, the impact of each module on improving model performance was analyzed.

Overall, compared to the SegFormer model, the improved modules in this study all contributed to enhancing model performance. Replacing the MLP layer of SegFormer’s decoder with the improved FPN allowed the integration of features at different scales, establishing connections among features of different scales. With an unchanged parameter count and computational complexity, the model’s PA, MIoU, and F1 scores increased by 0.18%, 0.25%, and 0.21%, respectively. The experimental results show that the Part-LKA module has a greater impact on improving model performance. By using depthwise and dilated depthwise convolutions, the module can effectively leverage both short- and long-range information, adapting well to both channel and spatial dimensions. Additionally, the $g^{n}$Conv module, with its spatial interaction properties, can effectively capture more detailed information, leading to better segmentation performance.

## 4. Discussion

Rice impurity segmentation faces various challenges. Firstly, environmental changes (e.g., lighting and weather) significantly affect the color and texture of rice and impurities, leading to unstable segmentation results. Secondly, the diversity and irregular shapes of impurities increase the complexity of model recognition. Overlapping and adhesion between impurities and rice make segmentation even more difficult, especially in complex images. In addition, impurities are often small, and detecting and segmenting small targets in large images reduces accuracy. Considering these issues, the performance of SegFormer often fails to meet the requirements of our subsequent research. Therefore, we enhanced the SegFormer model in several ways.

To improve the accuracy of the model when segmenting various impurities and enhance its representation capability, we replaced the MLP layer in the decoder with an improved FPN module and added Part-LKA and $g^{n}$Conv modules. Although this replacement significantly improved the model’s performance, it also increased the model size and computational complexity, necessitating further optimization.

Next, in order to deploy the algorithm on mobile embedded devices, we focused on lightweighting the model. We combined two 1 × 1 convolutions with the $g^{n}$Conv module to construct a Bottleneck-$g^{n}$Conv module, which reduced the model size and computational complexity through feature compression and increased network depth, while maintaining accuracy. Ultimately, a rice impurity segmentation model was built. Comprehensive comparisons with various mainstream models showed that the improved model performs excellently in terms of segmentation quality and model complexity.

In comparison with existing methods, our approach demonstrates several advantages. Jin et al. utilized an improved U-Net for soybean impurity segmentation, achieving high accuracy but encountering limitations in handling diverse impurity types and environmental conditions. Similarly, Liu et al. introduced a lightweight NAM-EfficientNetV2-based method, which improved segmentation efficiency but struggled with global feature representation. Unlike these CNN-based methods, our enhanced SegFormer leverages a Transformer-based architecture, providing superior long-range dependency modeling. Our method achieved a 1.6% increase in pixel accuracy and a 3.1% improvement in the $F_{1}$ score compared to the baseline SegFormer, while outperforming U-Net and NAM-EfficientNetV2 in both accuracy and computational efficiency.

In future research, we will first further improve the quality of the rice impurity dataset. We plan to capture and annotate images of various rice impurities from different angles and weather conditions to enhance the robustness of rice impurity segmentation in this study. Next, we plan to deploy the improved model on mobile embedded devices, and combine it with remote sensing technology to establish an automated and intelligent agricultural detection system. By analyzing the rice segmentation results in remote sensing images in real time, we aim to automatically detect anomalies, thereby achieving timely warnings and responses. This will significantly reduce agricultural risks and enhance the stability of rice production.

## 5. Conclusions

This study proposes a rice impurity segmentation model based on the SegFormer framework, with particular improvements made to its decoder. The original SegFormer decoder, composed entirely of MLPs, was overly simplified. Our hypothesis was that, by enhancing the representational capacity of the neural network and incorporating advanced feature fusion and attention mechanisms in the decoder, segmentation accuracy could be improved without significantly increasing model parameters and complexity, thereby not affecting its deployment on mobile devices. The experimental results support this hypothesis. The main findings are as follows:

1. The FPN module effectively fused high-level and low-level features, enriching the feature information;
2. The Part-LKA module successfully adjusted the feature intensity dynamically for each position and channel, emphasizing important spatial and channel features, thus enhancing the extraction of effective information;
3. The $g^{n}$Conv module significantly improved the representational capacity of the neural network, while the introduced Bottleneck-$g^{n}$Conv module effectively reduced model size and computational burden, maintaining high accuracy.

Comparison experiments indicate that the improved model outperformed the original SegFormer, with the pixel accuracy and F1 score improving by 1.6% and 3.1%, respectively. In addition, the model’s parameter count and computational complexity are 4.07 M and 4.66 G, respectively, and the model weight size is only 15.5M, making it suitable for deployment on mobile devices. This method provides an effective tool for rice impurity detection on mobile platforms.

## Figures and Tables

**Table 1:** Hardware and software parameters.

Environment	Item	Value
Hardware environment	CPU	AMD Ryzen 5 5600G
	GPU	NVIDIA GeForce GTX 1660s
	Video memory	6 GB
Software environment	OS	Windows 10
	Python	3.8
	Pytorch	1.12
	CUDA	11.6

**Table 2:** Comparison with other segmentation models.

Model	PA	mIoU	$F_{1}$	Params (M)	FLOPs (G)
NAM-EfficientNetv2	94.34	83.6	89.12	20.6	22.71
PSPNet	91.27	68.42	76.83	46.71	30.9
U-Net	92.48	71.05	78.77	24.89	113.08
DeepLabV3+	92.91	71.17	78.76	5.81	13.22
HRNet	92.69	71.38	78.99	9.64	9.37
SegFormer	96.17	83.76	88.47	3.72	3.39
Ours	97.77	88.82	91.57	4.07	4.66

**Table 3:** Training results of different attention modules.

Attention	PA	mIoU	$F_{1}$	Params (M)	FLOPs (G)
EMA	97.09	86.53	90.2	4.12	4.96
CoordAtt	96.31	84.14	88.78	4.73	5.1
SE	96.34	84.21	88.75	4.02	4.54
ECA	96.77	85.48	89.54	4.02	4.54
CBAM	96.5	84.78	89.14	4.34	4.85
Part-LKA	97.77	88.82	91.57	4.07	4.66

**Table 4:** Training results of different feature fusion modules.

Module	PA	GPA	SPA	BPA	$F_{1}$
NONE	96.17	97.35	79.7	83.6	88.47
FPN	96.18	97.36	79.9	83.78	88.49
U-net	94.34	96.23	65.09	78.83	83.4
SFM	91.88	95.21	64.87	78.15	80.32

**Table 5:** Ablation experiments of different modules.

Module	PA	mIoU	$F_{1}$	Params (M)	FLOPs (G)
SegFormer	96.17	83.76	88.47	3.72	3.39
+P-FPN	96.35	84.01	88.68	3.72	3.39
+P-FPN+Part-LKA	97.53	88.18	91.23	3.77	3.84
+P-FPN+Part-LKA+B-$g^{n}$Conv	97.77	88.82	91.57	4.07	4.66

## Data Availability

Data for this article can be obtained by contacting the corresponding author.
